# Sex and the TEs: transposable elements in sexual development and function in animals

**DOI:** 10.1186/s13100-019-0185-0

**Published:** 2019-11-03

**Authors:** Corentin Dechaud, Jean-Nicolas Volff, Manfred Schartl, Magali Naville

**Affiliations:** 1 Institut de Genomique Fonctionnelle de Lyon, Univ Lyon, CNRS UMR 5242, Ecole Normale Superieure de Lyon, Universite Claude Bernard Lyon 1, 46 allee d’Italie, F-69364 Lyon, France; 2 Entwicklungsbiochemie, Biozentrum, Universität Würzburg, Würzburg, Germany; 3 The Xiphophorus Genetic Stock Center, Department of Chemistry and Biochemistry, Texas State University, San Marcos, TX USA

**Keywords:** Transposable element, Sex determination, Sexual development and function, Germline, piRNA, Sex chromosome

## Abstract

Transposable elements are endogenous DNA sequences able to integrate into and multiply within genomes. They constitute a major source of genetic innovations, as they can not only rearrange genomes but also spread ready-to-use regulatory sequences able to modify host gene expression, and even can give birth to new host genes. As their evolutionary success depends on their vertical transmission, transposable elements are intrinsically linked to reproduction. In organisms with sexual reproduction, this implies that transposable elements have to manifest their transpositional activity in germ cells or their progenitors. The control of sexual development and function can be very versatile, and several studies have demonstrated the implication of transposable elements in the evolution of sex. In this review, we report the functional and evolutionary relationships between transposable elements and sexual reproduction in animals. In particular, we highlight how transposable elements can influence expression of sexual development genes, and how, reciprocally, they are tightly controlled in gonads. We also review how transposable elements contribute to the organization, expression and evolution of sexual development genes and sex chromosomes. This underscores the intricate co-evolution between host functions and transposable elements, which regularly shift from a parasitic to a domesticated status useful to the host.

## Background

Transposable elements (TEs) are major actors of the evolution of genomes and the diversification of species [1]. These DNA sequences have the peculiarity of being able to integrate into and spread within genomes, as well as to recombine and induce genome rearrangements, since they are generally repetitive. First discovered in maize [2], TE families described so far are generally divided into two main classes [3]. Class I TEs (retroelements) spread through a “copy-and-paste” mechanism called retrotransposition, which corresponds to a process of RNA-mediated duplication. They express an RNA intermediate that is reverse-transcribed into a cDNA fragment, which will be inserted somewhere else into the genome. Hence, retrotransposition directly increases the copy number of an element. In contrast, Class II TEs (DNA transposons) move through a “cut-and-paste” mechanism. Most autonomous class II elements encode a transposase that can bind to and excise the transposon from its initial genomic localization, and can subsequently insert it into a new locus [3–5]. This mechanism does not per se duplicate the initial transposon but only changes its location. However, the transposon can be duplicated if the transposition event occurs during the replication process, from an already replicated region to a non-replicated one.

Since they can insert into genomes, recombine and generate different types of rearrangements, TEs are by nature an important source of genomic variability between different species, or between individuals within a given species or population. Most insertions are thought to be deleterious for the host, in particular when they disrupt essential genes, regulatory regions or chromosomal structures, causing negative effects ranging from a slight decrease in host fitness to lethal mutations [6]. When a TE insertion is associated with such a fitness disadvantage, it is generally counter-selected and finally lost. The process of loss can however be modulated by several factors, including the selection coefficient of the insertion, its potential linkage disequilibrium with an advantageous allele, the recombination rate of the region of insertion, and the effective population size of the host [7]. Some insertions, in contrast, can be neutral, for example if they occur in genomic regions that have no crucial impact on host fitness, like gene-poor regions for instance. It is however difficult to classify an insertion as “neutral” once and for all, since it can still induce chromosomal rearrangements through ectopic recombination [8]. Lastly, some TE insertions might bring positively selected changes. In particular, TEs can spread ready-to-use regulatory sequences or trigger epigenetic modifications able to modify the pattern of expression of neighboring genes (for a review see [9]). TEs can also be “domesticated” as new host non-coding RNA genes or genes encoding useful proteins such as the syncytins, which are involved in the development of the placenta in mammals [10–12]. Syncytin genes have been repeatedly derived from *envelope* genes of endogenous retroviruses during mammalian evolution. Another example of TE-derived host proteins are the Rag proteins, which catalyze the V(D) J recombination responsible for the diversity of immunoglobulins and T cell receptors found in B and T cells, respectively. These proteins were formed from a Transib DNA transposon about 500 million years ago [13]. Many other examples of TE-derived genes have been described in different organisms (for a review see [11, 14]).

Persistence of TEs within a population, which would reflect their evolutionary success, requires their vertical transmission from one generation to the next. In animals with sexual reproduction, i.e. involving the fusion of male and female gametes, this implies transposition in the germline cells that will form the next generation. Sexual reproduction might be instrumental for the propagation of mainly deleterious TEs [15–17]. Indeed, in asexual populations, TEs might not be able to spread and tend to be eliminated if no horizontal transfer occurs [15–17]. Accordingly, experimental studies have shown that TEs are less fit to increase their frequency in asexual populations compared to sexual populations [15, 17–19]. Homologous recombination during meiosis is another feature of sexual reproduction that has an antagonistic impact on the fixation rate of TEs by favoring the elimination of deleterious TE insertions [20, 21]. Recombination triggers the exchange of genetic information between homologous chromosomes belonging to a same chromosome pair. This process has been associated to an increase of purifying selection since it drives the removal of deleterious point mutations and TE insertions [20, 21]. Hence, recombination and sexual reproduction could be considered as a defense mechanism against deleterious TE insertions. Reciprocally, high rates of deleterious mutations such as TE transpositions might favor the maintenance of sexual reproduction as an efficient way to keep these mutations at levels compatible with life [15, 17, 22–24]. In the asexual species *Leptopilina clavipes* (the wasp), no particularly high TE content is observed, despite the expansion of specific TE families, which could be linked to the switch toward asexuality [25]. The absence of recombination here does not seem to have triggered a massive expansion of TEs, or is counterbalanced by the limited spreading of TEs in the population due to asexuality. Similarly, no difference in TE composition was observed between the genome of an asexual fish of hybrid origin, the amazon molly *Poecilia formosa*, and the genomes of its parental sexual species, possibly due to the very recent occurrence of the switch from sexuality to asexuality in this lineage [26]. In the more ancient asexual taxa of the bdelloid rotifers, retrotransposons were long thought to be absent [27], supporting the role of sexuality in the genomic maintenance of these TEs [23]. More recent studies somehow challenged this model by highlighting a high diversity of TE families including LTR and non-LTR retrotransposons. However, each of these families presents a very low number of intact copies (one or two for the majority of them) [28]. Such a TE landscape, associated with the relatively low abundance of decayed fragments, the high similarity between LTRs for intact copies, and the localization of TEs in horizontally transmitted regions, led the authors to hypothesize that TEs were mostly acquired by recent horizontal transfers in rotifers [28].

In species with gonochoristic sex, i.e. species in which individuals are either male or female (in contrast to hermaphrodite species, in which individuals produce both male and female gametes), different factors can control sex determination (SD) [29, 30]. Some species undergo environmental sex determination (ESD), while others are subject to genetic sex determination (GSD). In ESD sex is determined by environmental factors, for instance temperature in turtles or crocodilians [31, 32]. Such temperature sex determination seems to be also present, albeit rare, in fish, as it was recently demonstrated for the Southern flounder [33]. In GSD on the contrary, the sex of the individual depends on its genotype. Sex can be determined by several interacting loci in a given species (polygenic sex determinism), but the most prevalent situation appears to be the monogenic GSD. In this situation, the chromosome pair that harbors the master SD gene becomes the sex chromosomes, or gonosomes. Two main sex chromosome configurations exist: the XX/XY system, particularly found in mammals, where males have two types of sex chromosomes (X and Y, male heterogamety), and the ZW/ZZ system, common in birds, where females have two different sex chromosomes (Z and W, female heterogamety) [34, 35]. Many other GSD systems have been reported such as haplodiploidy, where for instance males arise from haploid unfertilized eggs and female from diploid fertilized eggs, like in bees, ants, or some molluscs [36]. In the XX/XY sex determination system in mammals, the *Sry* gene is the male master sex determining gene for almost all species. *Sry* is located on the Y but not on the X chromosome and is therefore present in males but not in females. Non-mammalian species such as the fruit fly *Drosophila melanogaster* or the medaka fish *Oryzias latipes* also have XX/XY sex determination systems but of independent evolutionary origins. The *Sry* gene is absent from these species. In *O. latipes* the Y-linked master gene *dmrt1bY*, which is a Y-specific duplicate of the *dmrt1* gene, drives development toward the male phenotype like *Sry* in mammals [37, 38]. In *D. melanogaster*, the X chromosome carries *Sxl* that has to be in two copies to trigger female differentiation [39]. In this case, the initial choice between the male and female pathways is thus triggered by a dosage effect of the master gene. In birds, a similar process occurs but in a ZW/ZZ system, where ZZ males have two copies of the Z-linked *dmrt1* gene and females only one. This creates a gene dosage difference, leading to male or female differentiation [40]. In the nematode *C. elegans* individuals are either males or hermaphrodites. The presence of two X chromosomes (XX individuals) triggers the differentiation into a hermaphrodite adult that produces both male and female gametes. In contrast, XO individuals differentiate into males as a consequence of the ratio between X chromosomes and autosomes [41, 42].

Once sexual development is initiated, the gonad, which comprises both germ cells and somatic cells, differentiates into either a testis or an ovary. A sex-dependent gene regulatory cascade, initiated in the somatic part of the gonad, controls differentiation [30, 43, 44]. Male and female differentiation cascades are often repressing each other, creating a competition between male and female differentiation genes: the most expressed pathway represses the other one [43]. Finally, once the gonad is differentiated, sex is maintained by the expression of specific genes like those encoding the sexual hormone biosynthesis pathways in mammals. It has been shown in mammals and teleost fish that even in adults, de-repressing the opposite pathway can induce sex reversal [45–47]. This demonstrates that expression of at least some of the sexual development network genes is necessary to maintain the differentiated state in sexually mature individuals. Beyond gonads, sex affects many other pathways in the organism, creating a bias in gene expression in several tissues and organs including brain [48–53]. However, gonads remain the most sex-biased organs in terms of gene expression.

Depending on the animal lineage, sexual development and particularly sex determination can show very different evolutionary dynamics. Some SD systems are ancient and at least 100 million years old, such as the mammalian male heterogamety system driven by the Y-linked gene *Sry* [54] or the avian female heterogametic determination controlled by the Z-linked *dmrt1* gene [40]. In other lineages, for instance in teleost fish, sex determination is much more labile, with a frequent switch between and even combination of ESD and GSD, and an important turn-over of sex chromosomes and master sex-determining genes in GSD [55, 56]. For example, the genetic sex determination system is not conserved in the genus *Oryzias*: while *O. latipes*, *O. curvinotus*, *O. luzonensis* and *O. dancena* use a XX/XY system, *O. javanicus* determines sex through ZW/ZZ female heterogamety [57]. Strikingly, Oryzias species with a XX/XY system generally have different sex chromosomes and even different master sex-determining genes: sex is controlled by *dmrt1bY* (aka *dmy*) in *O. latipes* and *O. curvinotus, gsdfY* in *O. luzonensis* and *sox3Y* in *O. dancena* [57]. Hence, the control of sexual development can be considered as a fast-evolving trait in this clade. Beyond the initiation of sex differentiation, the downstream molecular pathways also appear variable among animals: a comparison of genes expressed in medaka fish and mammalian gonads revealed substantial differences [58]. Very interestingly, the control of sexual development sometimes experiences convergent evolution: in both therian mammals (non-egg-laying placental mammals and marsupials) and *Oryzias dancena* for instance, the master sex-determining gene evolved from the *Sox3* gene [59]. This happened independently in the two lineages, 148 to 166 million years ago in a common ancestor of therian mammals, and less than 20 million years ago in *Oryzias dancena*. Another striking example is the *dmrt1* gene in birds and in the tongue sole. This gene was ancestrally located on the vertebrate linkage group A, which became the Z chromosome independently in both lineages [60].

In this review, we reassess the impact of transposable elements on the structure and expression of genes and genomes through the prism of sex by inventorying the known reciprocal interactions between TEs and sexual development and function in animals. The species sample, however, appears heavily biased towards insects and vertebrates, since most of the studies linking TE and sex have been conducted in classical model organisms commonly used in genetics and development. We first focus on the expression of TEs in germ cells and on the control of their expression. Then, we review how TEs, reciprocally, can impact the expression of sexual development genes. Finally, we document how TEs influence the organization and structural evolution of sexual genes and chromosomes. These diverse and reciprocal influences well illustrate the intricate co-evolution of TEs with their host.

## TE expression is tightly controlled in the germline

### TEs in the germline: a trade-off between expression and control

Expression and transposition of TEs in the germline are necessary for their vertical transmission to the host progeny, and ultimately for their maintenance within a lineage. The first step of TE transposition consists in the transcription of mRNA to produce enzymes such as a transposase for most DNA transposons, or a reverse transcriptase and an integrase/endonuclease for retroelements. TE mRNAs are expected to be found in cells where TEs are spreading. TE-derived transcripts are indeed found in transcriptomes [61–64], including the germline [65, 66]. In the medaka *Oryzias latipes* for instance*,* about 1.2 and 3.5% of the transcriptome of ovaries and testes, respectively, can be assigned to TEs (Dechaud et al. unpublished data).

If evolution fosters TEs that are active in gonads, the putative negative effects of TE insertions, at the same time, require repressive mechanisms. The gonadal activity of a TE results in a trade-off, its own survival depending on the survival of the host, which is needed for vertical transmission and maintenance. This follows the “selfish gene” hypothesis according to which, in a gene-centered view of evolution, some genes can enhance their own transmission, sometimes with a negative effect on the organism fitness [16]. Very interestingly, some TEs like the P element in Drosophila produce different transcripts depending on the organ in which they are expressed [67]. In the gonads, the third intron of the P element is excised allowing its transposition, while in the soma, in addition to a transcriptional control, the P element transcript keeps its third intron and is not able to transpose [67]. Such mechanisms allow the element to limit its impacts on the soma while transposing in the germline.

### Germline TE expression is controlled by several mechanisms

#### piRNAs (Fig. 1a)

Piwi-interacting RNAs (piRNAs) are 24–31 nucleotides long small non-coding RNAs expressed in the germline and derived from long RNAs that contain TE sequences [68]. They have been described in eukaryotes only, from humans to protozoans [69, 70] and play a large diversity of roles, such as genome rearrangement in ciliates, sex determination in silkworm, telomere protection in Drosophila, long-term memory in sea slug, or oocyte development in human [70]. piRNAs are produced from specific loci called piRNA clusters that regularly integrate new TE-derived sequences and thus extend their target potentialities. They can further be amplified by the so-called “ping-pong” cycle [71].

**Fig. 1:**
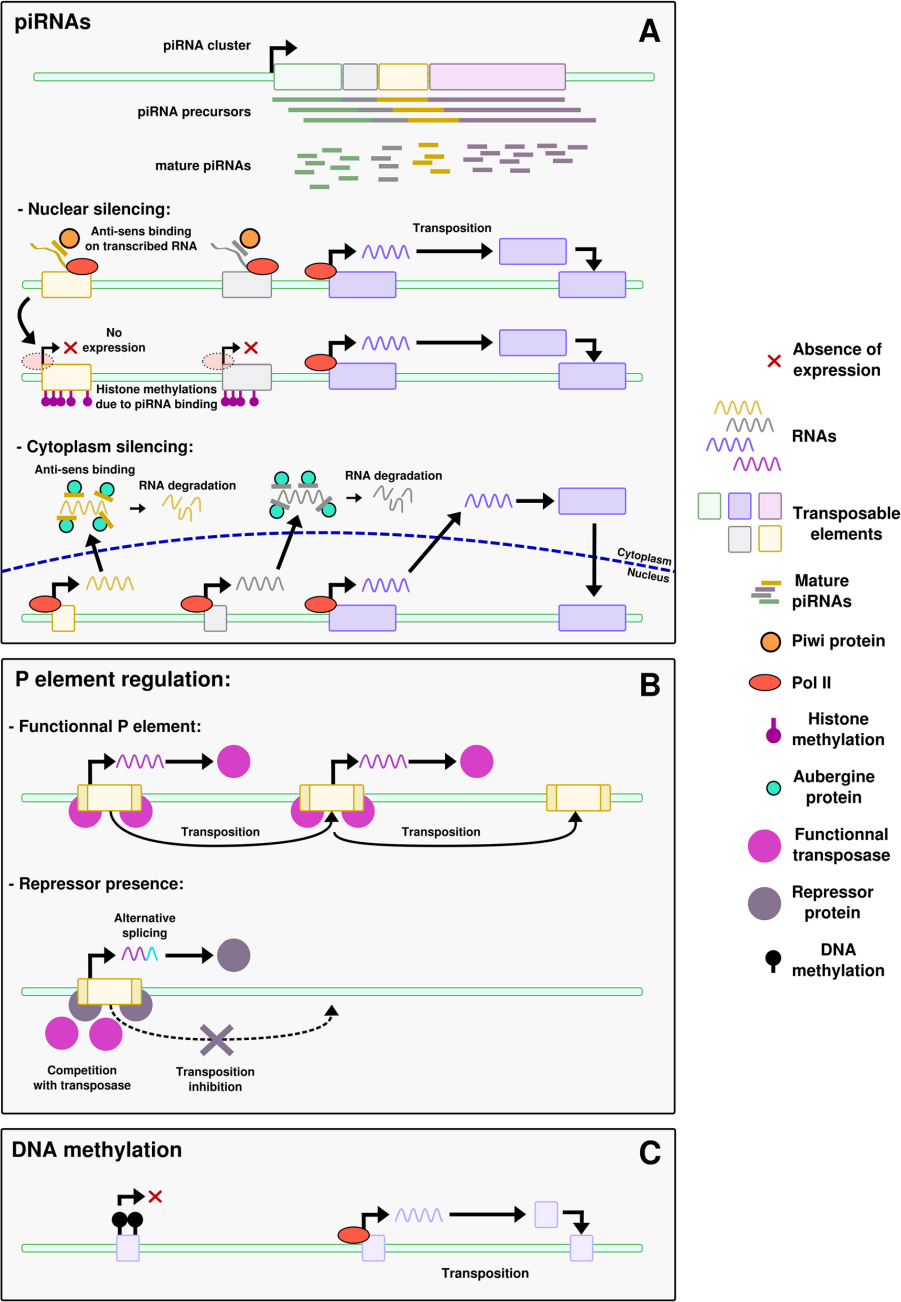
Different ways to control TE expression. **a** piRNAs. piRNAs are produced from piRNA clusters, genomic spots where new TEs can integrate. piRNAs can act through two mechanisms. In the nucleus, piRNAs bind to Piwi proteins. They also bind in anti-sense to TE mRNA being transcribed, triggering histone methylation of TEs and thus inhibiting recruitment of Pol II. This leads to the silencing of TE expression. In the cytoplasm, piRNAs bind to other Argonaute proteins, triggering TE mRNA degradation. **b** Repressor proteins. A functional P element produces the transposase that triggers its excision and transposition. When repressor proteins are transmitted from the mother through cytoplasm or when the P element is degenerated, it produces an alternatively spliced mRNA. This mRNA encodes a non-functional transposase that will act as a repressor by competing with the functional transposase, and trigger the production of more alternatively spliced mRNA. This positive repression loop, where the repressor protein activates its own production, prevents the transposition of the TE. **c** DNA methylation. The TE is methylated, preventing its expression

piRNAs can regulate TE expression by two different mechanisms. The first mechanism occurs in the nucleus, where piRNAs interact with the Piwi proteins, a subfamily of Argonaute nucleases, to target the TE nascent RNAs to which they present sequence similarities, and adds histone repressive marks in the region by interacting with other proteins [68]. This mechanism inhibits the expression of the targeted TEs. The second mechanism happens in the cytoplasm, where piRNAs form a complex with Aubergine (Aub) proteins, which belong to the Piwi subfamily too. This complex post-transcriptionally silences TE expression by interacting with the TE mRNAs. This also triggers a replication of the piRNA, known as the ping-pong cycle [68]. The ubiquitous presence of this regulatory system in the gonads specifically underlines the importance of controlling TE activity in the germline.

As an example, piRNAs are involved in the P-cytotype regulation in *Drosophila* [72]. In these species, some strains of flies have a DNA transposon, the P element, from which a complementary piRNA is produced. These are called “P strains”, for Paternal contributing strains, in opposition to “M strains”, for Maternal contributing strains. One model proposes that in P strains, P element-derived piRNAs are transmitted from the mother through the oocyte cytoplasm. The transmitted piRNAs then silence the P element both in the nucleus and the cytoplasm by the mechanisms described above. piRNAs are further amplified in the cytoplasm through the ping-pong cycle, maintaining the silencing of the P element. If no piRNA is transmitted from the mother, the P element is not repressed. Consequently, a P male crossed with an M female will have a dysgenic offspring, with increased mutation rates, frequent sterility and abnormally small gonads [73]. This phenomenon, due to the fact that the offspring have the P element but no silencing through maternal piRNA, is known as “hybrid dysgenesis” [67, 72]. In contrast, the offspring of a P female crossed with an M male is fertile, as the P female brings the P element but also some piRNAs to trigger its repression, as well as the ping-pong amplification cycle.

#### Repressor proteins (Fig. 1b)

TE expression can also be directly controlled by protein factors. In vertebrates, KRAB-ZNF (for Krüppel-associated box domain zing finger) proteins have been shown to play this role ( [74], reviewed in [75]). They constitute a large family of proteins and are able to bind various DNA sequences via the diversity of their ZNF domains. They recruit KAP1 (for KRAB-associated protein 1) to DNA, which in turn mediates transcriptional silencing through histone modifications. KRAB-ZNF proteins were first discovered in mice where they silence genomic insertions of a murine leukemia virus (MLV) [76], but recent studies demonstrated their action on other retroelements [77]. Many KRAB-ZNF proteins are expressed during germline development; however the targeted TE families are still to be discovered for most of the KRAB-ZNF members [77–79]. In Drosophila, a second model of P-element control involves repressor proteins. P strains express a repressor protein that prevents the transposition of the P element in the germline. This mechanism is known as the “protein repressor model” [67, 72]. The repressor is produced from degenerated P elements or from alternatively spliced full P element transcripts. If the precise action mechanism of the repressor protein is unknown, the main hypothesis is a competitive inhibition with the P element transcription [72]. This repressor could also further trigger the production of alternatively spliced transcripts, leading to a feedforward repression loop (Fig. . 1); however this action as a splicing modifier has never been demonstrated. It is inherited from the mother through the cytoplasm. Since the discovery of piRNA however, later demonstrated to repress TEs in the germline [80], an alternative model has been proposed for the P-cytotype regulation (see before). Both models are not mutually exclusive and likely coexist within populations or individuals [72].

#### Epigenetic modifications (Fig. 1c)

TE activity can be controlled by epigenetic regulations such as DNA methylation [9] or histone modifications [80, 81]. These epigenetic controls however are not specific of the germline. The modifications targeting TEs can sometimes also affect neighboring genes, hence participating in shaping their regulation and influencing genome evolution [82]. Indeed, the epigenetic silencing of TEs is known to be released in cases of stress, for example UV exposure or temperature changes [83]. Thus TEs can be reactivated and expand, influencing genome evolution under stress conditions [82].

### TE expression can vary between sexes

Epigenetic modifications and gene expression can differ between sexes. One may wonder, because of these epigenetic differences, whether TE activity would also vary between males and females. Some TE families are expressed at unchanged levels in very different contexts, like SINEs in rats [84]. In this study, 11 organs were tested including testis and uterus, each at 4 developmental stages. Contrary to SINEs, LTR appeared to be more likely to be expressed in specific tissues or conditions, and are also found more differentially expressed between sexes [84, 85].

In mammals, the inactivation of the Piwi regulatory system in the germline of males leads to azoospermia (no production of mature gametes) due to a high rate of illegitimate pairing between non-homologous chromosomes at meiosis that trigger apoptosis [86]. Also, piRNA interacting protein expression was found to be impaired in humans with cryptorchidism (absence of both testes, or location outside the scrotum) [87]. In contrast, Piwi system inactivation in female mice does not lead to over-activation of TEs [86], and neither does a knock-out of *dicer*, a protein involved in the siRNA degradation system, which would have suggested the involvement of the RNA interference pathway in TE control. One player of this control corresponds instead to the evolutionarily conserved MAEL protein (encoded by the *maelstrom* gene), found both in mouse and fly [88]. When this factor is mutated, a 2.3-fold excess of L1 mRNA is measured in embryonic day 15.5 mouse oocytes [88]. Although its precise role is still unclear, MAEL intervenes in a silencing step downstream of Piwi [64]. Of note, TEs are hypomethylated in females compared to the male germline. Hence, oocytes seem more resilient to TE transposition than the male germline. It has been suggested that this difference could be linked to the life-long division of spermatogonial cells, in contrast to oocytes, which undergo a long meiotic arrest. Cell division is required for TE transposition, and many more cell divisions occur in the male germline. More cell divisions would allow too many deleterious insertions in the male germline, explaining the need for TE silencing [86].

## TEs can regulate the expression of sexual development genes

TEs can have an important impact on gene regulatory networks [89–91]. They can modify the expression of surrounding genes [9, 91] by bringing with them Pol II or III promoters as well as transcription factor binding sites, insulators, splicing sites or epigenetic modifications. TEs could be particularly prone to recruitment into sexual development since they are generally expressed in the gonads.

### *Regulation in cis* (Fig. 2a)

TEs have a strong cis-regulatory potential for host genes through their Pol II or Pol III promoters and binding sites for transcription factors, or other regulatory sequences, which they carry [9]. These regulatory sequences can already exist in the TE sequence, or derive from this sequence by a few point mutations only. Some of the described examples are related to sexual development.

**Fig. 2:**
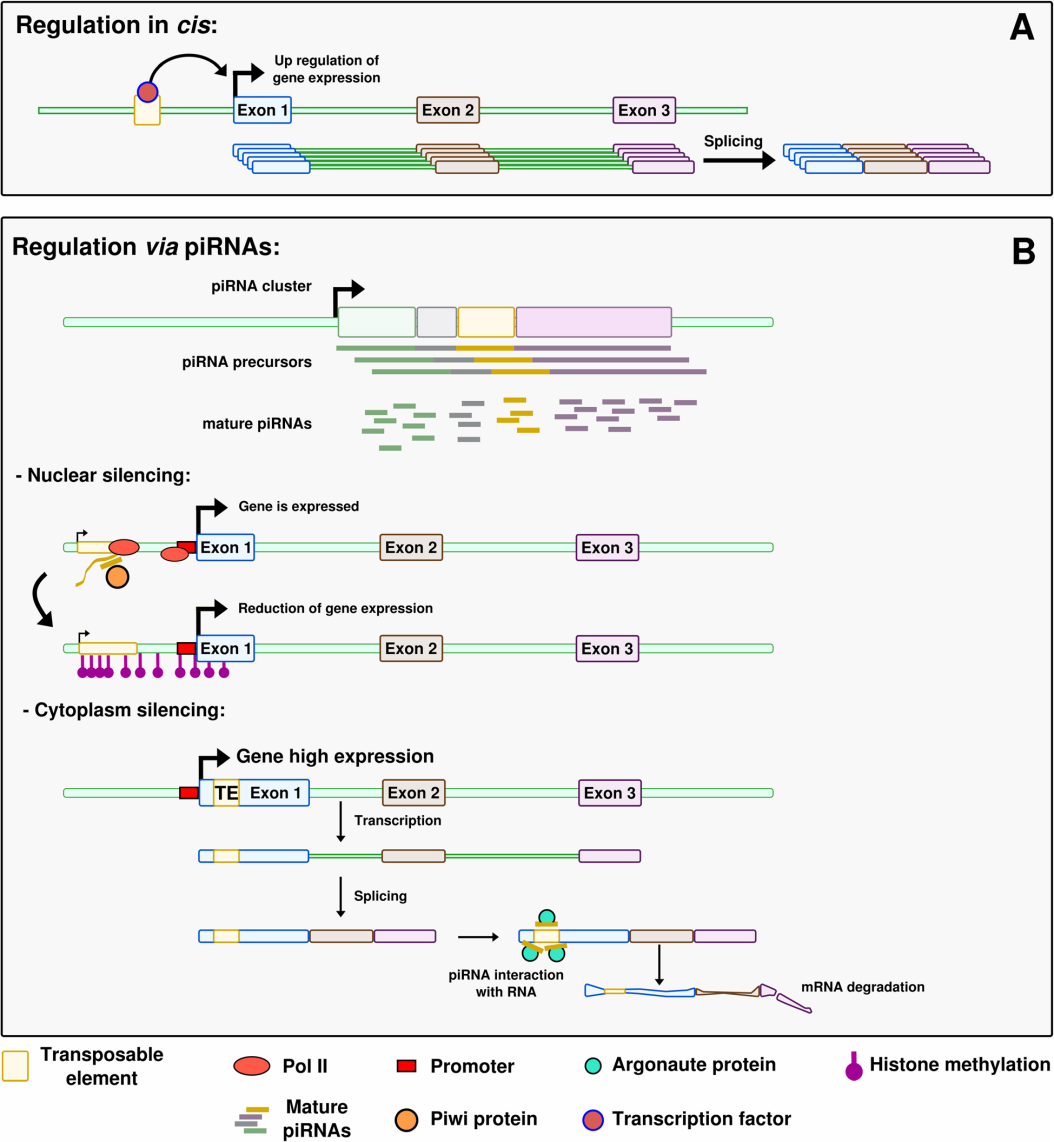
Different ways how TEs can affect gene expression. **a** Regulation in *cis*. The TE brings a ready-to-use regulatory sequence that carries a transcription factor binding site. The transcription factor can bind on this site and influence expression of the neighboring gene. **b** Regulation via piRNAs. In the nuclear silencing situation, a TE is present close to the gene of interest. The piRNA, via the Piwi protein, triggers histone modifications that silence the TE but also affect the RNA polymerase binding region of the neighboring gene. Because of the epigenetic modification of the TE, the gene expression is reduced. In the cytoplasm silencing situation, a TE-derived sequence is present in the 5’UTR of the gene. piRNAs specific to this TE bind the transcript in the cytoplasm via an Argonaute protein and trigger the degradation of the transcript

In Drosophila species, MSL Recognition Elements (MREs) are known to trigger dosage compensation for X chromosomal genes. MSL (for Male Specific Lethal) is a male-specific complex that binds to MREs and increases neighboring gene expression in XY males, hence compensating for the absence of one X chromosome compared to XX females. MREs are found at multiple loci interspersed on the X chromosome. Interestingly, they are carried by Helitron DNA transposons that regulate in *cis* genes close to their insertion sites [92, 93]. In *Drosophila miranda* the X chromosome is recent, allowing the detection of the Helitron sequences with alignments methods, while in other *Drosophila* with older X chromosomes, MREs are present but the Helitrons are not detectable anymore. The authors propose that, on these older chromosomes, selection eroded the Helitron TEs outside of the selected MRE motifs [92, 93]. This example illustrates the efficiency of TEs in the rewiring of gene regulatory networks, as they can spread transcription factor binding sites or other types of regulatory sequences that can then co-regulate several genes. This process appears even more efficient than the birth of transcription factor binding sites “from scratch” by a series of point mutations, which would require much more time to target different genes [89]. More recent studies on MSL in *Drosophila* show that other mechanisms such as microsatellites expansion also spread MRE motifs on neo-X chromosomes [94]. In *Drosophila melanogaster*, the promoter of the *Su (Ste)* piRNA – one of the most abundant piRNA in the testes – derives from a *1360* transposon [95, 96]. *Su (Ste)* silences the *Stellate* genes, hindering the accumulation of *Stellate* proteins, which causes formation of crystals and results in male sterility [97].

**Fig. 3:**
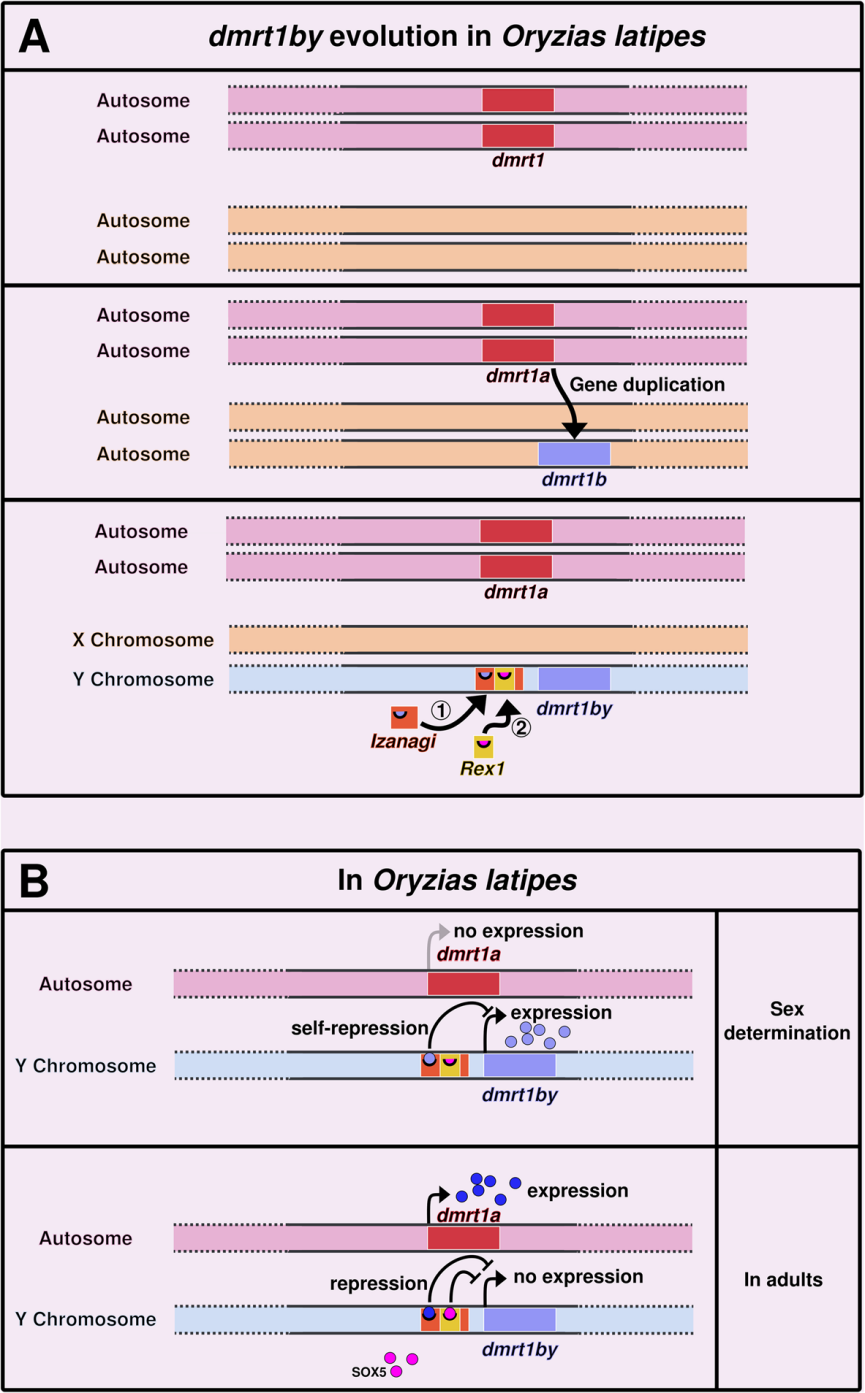
dmrt1bY evolution and regulation in *Oryzias latipes.* **a** In the ancestor, the *dmrt1* gene existed in a single copy on a pair of autosomes. *dmrt1* was then duplicated into *dmrt1a* and *dmrt1b*. Later, two TEs inserted upstream of *dmrt1b*: *Izanagi,* a DNA/P-element, followed by *Rex1,* a LINE retrotransposon [98]. After the insertion of these TEs, *dmrt1b* became the master sex-determining gene *dmrt1bY* and the chromosome harboring it became the Y chromosome (the gene is absent from the X). **b** *dmrt1bY* is expressed during sex determination in the prospective males. Its product triggers sex determination towards the male phenotype. It also binds on its own binding site in *Izanagi,* down-regulating its own expression. After sex determination and in adults, *dmrt1a,* the ancestral paralog of *dmrt1bY*, is expressed. It binds to *Izanagi*, down-regulating and silencing *dmrt1bY* once sex determination has occurred. This silencing is also ensured by the binding of Sox5 to a motif encompassed in the *Rex1* sequence

Other cases of TE-controlled genes have been described in other organisms. In the medaka fish *Oryzias latipes,* the master sex determining gene *dmrt1bY* has been formed through the duplication of the autosomal gene *dmrt1a,* which has a downstream position in the male sex differentiation cascade in vertebrates. *Dmrt1bY* is controlled by different transcription factors including itself, its paralog Dmrt1a and Sox5. Binding sites for these transcription factors are located in the upstream region of *dmrt1bY*, which corresponds to a non-autonomous P element called *Izanagi*, in which a LINE/Rex1 retroelement was inserted later (Fig. 3a) [98]. The binding sites for Dmrt1A and Dmrt1bY are located within *Izanagi,* while the binding site for Sox5 lies within the Rex1-derived sequence [47, 98]. Here, the TEs directly brought the *cis*-regulatory elements that conferred to *dmrt1bY* an expression pattern compatible with a function as a master sex-determining gene. This makes a convincing case for TEs being actors of sex determination evolution (Fig. 3b) [98]. Accordingly, it has also been suggested that recent TE insertions in humans (like *Izanagi* in medaka) usually bring context-specific gene activities, while older TE insertions are more likely to correspond to broad enhancers [99]. In human, enhancers are globally depleted in recent TE insertions. However, enrichment of young TE families is observed in enhancers of genes specifically expressed in testis [99].

### *Regulation by piRNAs* (Fig. 2b)

TEs can affect the regulation of genes in *trans* via piRNAs. If piRNAs are originally devoted to the down-regulation of TEs, there is now accumulating evidence that piRNAs regulate host developmental genes and maternal mRNA decay [100]. As an example, TE-derived piRNAs can target maternally-deposited copies of the Drosophila embryo *nos* mRNA for degradation, which is required for a proper development of the head [101]. The region of the *nos* 3′ untranslated region that is recognized by the piRNAs originates from two different TEs [101]. We can find some evidence of such regulation in gonads. In Drosophila ovarian somatic sheet cells a piRNA knock-down affects the expression of about 100 transcripts [102]. Most of these deregulated transcripts originate from TEs, but a significant part of them still corresponds to host protein-coding genes, with different genes being affected according to cell lineage. Some of these genes presented de novo inserted TEs in their introns or UTRs that induced suppression by the PIWI machinery at the nascent RNA level [102]. In mouse spermatocytes, piRNAs derived from TEs were shown to mediate the degradation of numerous mRNAs and lncRNAs [103]. This regulation involves PIWIL1, a major actor of the piRNA pathway, the knockdown of which leads to the upregulation of 172 genes. piRNAs were shown to target in particular retrotransposon sequences located in the 3′ UTR of mRNAs [103]. TE-derived sequences thus play a role in the control of germline expressed genes through piRNAs.

Some piRNAs have been demonstrated to trigger sex determination. In *Bombyx mori*, a species where the sex determining system is ZW/ZZ, the master sex-determining region is localized on the W chromosome and produces female enriched piRNAs deriving from TEs and repetitive sequences. The *Fem* piRNA encoded in this sex-determining region of the W chromosome derives from a non-TE repetitive region and forms a complex with a silkworm equivalent of the Piwi protein. The complex targets and cleaves a masculinizing protein-coding mRNA transcribed from the Z chromosome, triggering feminization [104, 105]. A similar example has been described in *C. elegans*, where the *21ux-1* piRNA downregulates the *xol-1* gene involved in X chromosome dosage compensation and sex determination [42]. This piRNA control of *xol-1* appears to be conserved in the related nematode *C. briggsae*, suggesting a robust involvement of piRNA in controlling gene expression [42]. In these two examples however, neither the piRNA nor its target were shown to be derived from TEs. In mammals, as described before, the inactivation of the epigenetic control of TEs in male gonads leads to azoospermia and thus infertility [86]. However, a certain relaxation of epigenetic control is observed in the germline, leading to demethylation of TEs and their reactivation. At a first look, this could be considered as deleterious for the host. The relaxation happening in the germline leads to a low level of TE activity that is actually thought to allow the host to sense the TEs present in the genome [86]. Such sensing would help to better control TE transposition. According to the authors, this sensing could be ensured by piRNAs. Relaxation of the epigenetic control allows TE expression that itself triggers piRNA production. piRNAs could then limit the impact of TEs but also regulate the expression of other genes, and through these possibly participate in sexual development. Taken together, the presence of TEs in genomes could be linked to the fact that they have an indirect effect, via piRNAs, on the control of specific genes, and sometimes on critical event such as sexual development.

## TEs are involved in sex chromosome structure and evolution

We have described how sex can influence TEs expression, and reciprocally how TEs can modulate expression of genes involved in sexual development. In addition to effects of TE on host gene expression, genomic differences can exist between males and females in terms of TE and gene position and content. These differences can impact sexual development.

In mammals, the X and Y chromosomes are derived from a same pair of autosomes. Accordingly, even if the Y chromosome has lost many of its genes due to suppression of recombination, most genes carried on the Y chromosome have homologs on the X chromosome. This scenario of gene loss, however, does not appear universal, since in certain cases, like in *Drosophila melanogaster*, sex chromosomes evolved more through gene gain [106]. In the platyfish (*Xiphophorus maculatus)*, an accumulation of *Texim* genes is observed on the Y chromosome [107]. These genes are physically associated to a Helitron transposon, which might have spread the *Texim* sequences on the Y chromosome but not on the X. In salmonids, recent findings on SD showed that the master sex-determining gene, *sdY*, is conserved in many species. However, it does not always locate on the same chromosome, but instead seems to behave like a “jumping gene” [108, 109]. An analysis of the boundaries of the moving region that carries *sdY* revealed the presence of several TE sequences, leading authors to propose a mechanism of TE-associated transduction [108, 109]. This phenomenon could be linked to a rapid turnover of sexual chromosomes in this clade. Other examples of such sex determining “jumping genes” have been described in animals, such as in the house fly [110] or in *Chironomus* species [111]. In these cases the possible involvement of TEs in the translocation of the determining cassette has not been investigated, but we can notice that, in the case of the house fly, about two thirds of the Y-linked scaffolds present sequence similarities with TEs [110].

TEs can also themselves present sex-specific localizations. As described before, in *Drosophila miranda* the recently formed X chromosome, called “neo-X”, accumulates Helitron DNA transposons [92]. The success of fixation of this TE on this specific sex chromosome is probably linked to its role in the expression of X-chromosomal genes, bringing an evolutionary advantage (see part 2A) [92]. Sex chromosomes are actually often enriched in TEs [112–115]. This accumulation might be in some cases the consequence of the impossibility for sex chromosomes to recombine and thus eliminate deleterious insertions. In the genome of the African clawed frog *Xenopus laevis,* recombination between W and Z sex chromosomes stopped recently, and a large accumulation of TEs already started in the W specific regions [115]. Such accumulation has also been observed on several young sex chromosomes of teleost fishes [112]. The higher density of TEs on these chromosomes might increase their probability to regulate some key sexual development genes and consequently to impact sexual development. In birds, such as woodpeckers for instance, the female specific chromosome W is enriched in CR1 insertions, which is a retrotransposon [116, 117]. In human, the Y chromosome is a hot spot for specific TE insertions [118]. All TE types show a higher density on the Y compared to autosomes, except for SVA short retrotransposons. In particular, density is 30 times higher than the genome average for LTR elements, and four times higher for *Alu* and L1 elements. The authors assume that this cannot be due to a genome assembly artifact, since the enrichment varies according to TE families. Nevertheless, they do not provide any explanation for the insertion rate differences between TE types on the Y chromosome. This high TE density on the Y chromosome is not explainable by low gene density as human chromosome 13 has a lower gene density and is not enriched for TEs [118]. This accumulation of active elements suggests that the Y chromosome is not shrinking in man, but still expanding through new insertions [119]. Of note, in contrast to what is observed in mammals and birds, the heterogametic sex chromosome (W or Y), in many fish, reptiles and amphibians, is much larger than the Z or X, and often the largest chromosome of the complement. In these groups, sex chromosomes are usually younger than in mammals and birds, with frequent turnover. In addition to bringing additional DNA material, it has been hypothesized that TE insertions could favor, in a fast and effective manner, structural differences between gonosomes, that in turn help the expansion of the region of suppressed recombination [120]. This could thus lead to an increase in sex chromosome size during the early phase of their differentiation, while size diminishing would occur later in their evolution [120]. The accumulation of TEs and other repetitive sequences on the Y chromosome has been hypothesized to globally impact the chromatin landscape of the genome [121, 122]. Indeed, polymorphic Y chromosomes that differ only by their quantity of repeats are associated to different levels of chromatin repression on autosomes [122]. The high density of TEs and satellite DNA on the Y chromosome could function as a sink for heterochromatin marks, leading to a dilution of these marks in the rest of the genome, and hence to differential expression between males and females [122].

The X chromosome inactivation in mammals, also called Lyonisation, is a dosage compensation process in which one of the two X chromosomes is inactivated in XX females, preventing gene overexpression compared to males, which have a single X [123, 124]. The enrichment of LINE retrotransposons on the X chromosomes of human and mice led to the hypothesis of an involvement of LINEs in this process [114, 124]. This hypothesis has been tested in the spiny rat *Tokudaia osimensis*, where males and females are XO [125]. No dosage compensation by X inactivation is required here, suggesting that LINEs would not be required on this X chromosome. Interestingly, the authors describe a similar high concentration of LINEs on this X chromosome compared to humans or mice. They conclude that the accumulation of TEs on X chromosomes might be only a by-product of reduced recombination [125]. This idea was also reviewed later by Lyon, leading to the same conclusion [126]. Further investigations on the role of LINEs in X chromosome inactivation have been conducted in mammals. On the human X chromosome, regions poor in L1 elements contain genes escaping X inactivation [127]. In placental mammals, the inactivated X chromosome is coated with Xist (X-inactive specific transcript) RNAs, which have a silencing effect. These regions are composed of silent LINEs that are closed in chromatin 3D structure, and are formed prior to gene inactivation [128, 129]. As genes “move” in the Xist silenced region via a modification of the 3D conformation of the chromosome, they become inactivated. Conversely, LINE poor regions are physically distant from Xist silenced regions [123, 129]. In these studies, the authors show that LINEs play a role in the spread of X chromosome silencing by recruiting Xist RNAs, suggesting a general role in the regulation of X-chromosomal gene expression. This phenomenon also exemplifies that for understanding chromosomal organization the intricate structure and function relationships have to be considered.

## Conclusions

Sex is an important parameter to take into account when performing experiments, in particular when analyzing gene expression [130]. Many studies, including genome sequencing, are conducted in individuals of only one sex, and results observed might not be generalizable to the other [131]. We presented in this review the many facets linking sex with TEs, both influencing each other in a co-evolutionary process. TE expression in germlines is essential for them to get fixed in the genome and be transmitted vertically. Conversely, TEs have an influence on sex differentiation mechanisms, for example through the intermediary of piRNAs. They could also influence sex evolution by the regulatory novelties they create. TEs are indeed great tools for evolution as they can rapidly propagate regulatory elements and thus provide the necessary rewiring of the genetic network. The high density of TEs on sex chromosomes, linked to the absence of recombination of these chromosomes, could increase the probability for TEs to locate in the vicinity of sexual development genes and interact with them. They can influence and be influenced by sex depending on the process studied.

Another way TEs can influence gene expression is by triggering alternative splicing, via the new splicing sites they sometimes bring with them [9]. In the case of sexual development gene regulation, however, such involvement of TEs has yet to be demonstrated. In *Drosophila melanogaster*, some intron retention events are known to be linked to sex [132]. Although the exact trigger of the alternative splicing is not clearly elucidated for now, a hypothesis proposed that the high coverage of repetitive sequences on the Y chromosome could be involved in the process, as presented earlier in this review: the Y chromosome would attract on its repeats high quantities of chromatin-modifying proteins, which would in turn lead to a global modification of the chromatin state on other chromosomes, and in the end would affect the accessibility of splicing factors to the nascent transcripts. Here, the impact of TEs on the splicing machinery would thus be indirect and not specific to particular genes.

Finally, genes involved in sexual development and sexual functions seem to evolve faster than other genes [133, 134]. These observations of positive selection and rapid evolution are not really consistent with earlier observations of the sex determination and differentiation cascade. Indeed, a popular model, formulated by Graham in 2003, states that “masters change, slaves remain” [135], where “masters” refer to genes at the top of the sex determination cascade, and “slaves” to genes acting at the end of the cascade. A renewal of this initial proposition has been proposed by Herpin et al.: “When masters change, some slaves remain, others are dismissed or acquire new tasks, and new ones can be hired” [34, 55]. Knowing that TEs are a source of genomic diversification, studying the evolution of sexual development genes in the perspective of TEs, just as the evolution of their regulation, could reveal interesting trends. A perspective could be to investigate RNA-seq dataset for species-specific sex-biased genes associated to TE location variation between closely related species to reveal candidate genes recently controlled by TEs. Global approaches by sequencing piRNAs and mapping them to sex-biased genes could also give more clues about the regulation and evolution of genes involved in sexual development and function.

## Data Availability

Data sharing not applicable to this article as no datasets were generated or analysed during the current study.

## Abbreviations

ESD: Environmental Sex Determination
GSD: Genetic Sex Determination
KAP1: KRAB-associated protein 1
KRAB-ZNF: Krüppel-associated box domain zinc finger
MRE: MSL Recognition Element
MSL: Male Specific Lethal
piRNA: Piwi-Interacting RNA
SD: Sex Determination
TE: Transposable Element
